# Transcriptionally Repressive Chromatin Remodelling and CpG Methylation in the Presence of Expanded CTG-Repeats at the DM1 Locus

**DOI:** 10.1155/2013/567435

**Published:** 2013-12-23

**Authors:** Judith Rixt Brouwer, Aline Huguet, Annie Nicole, Arnold Munnich, Geneviève Gourdon

**Affiliations:** ^1^Inserm U781, Hôpital Broussais, Batiment Leriche, porte 9, 96 rue Didot, 75014 Paris, France; ^2^Université Paris Descartes-Sorbonne Paris Cité, Institut Imagine, 156 rue de Vaugirard, 75015 Paris, France

## Abstract

An expanded CTG-repeat in the 3′ UTR of the *DMPK* gene is responsible for myotonic dystrophy type I (DM1). Somatic and intergenerational instability cause the disease to become more severe during life and in subsequent generations. Evidence is accumulating that trinucleotide repeat instability and disease progression involve aberrant chromatin dynamics. We explored the chromatin environment in relation to expanded CTG-repeat tracts in hearts from transgenic mice carrying the DM1 locus with different repeat lengths. Using bisulfite sequencing we detected abundant CpG methylation in the regions flanking the expanded CTG-repeat. CpG methylation was postulated to affect CTCF binding but we found that CTCF binding is not affected by CTG-repeat length in our transgenic mice. We detected significantly decreased *DMPK* sense and *SIX5* transcript expression levels in mice with expanded CTG-repeats. Expression of the DM1 antisense transcript was barely affected by CTG-repeat expansion. In line with altered gene expression, ChIP studies revealed a locally less active chromatin conformation around the expanded CTG-repeat, namely, decreased enrichment of active histone mark H3K9/14Ac and increased H3K9Me3 enrichment (repressive chromatin mark). We also observed binding of PCNA around the repeats, a candidate that could launch chromatin remodelling cascades at expanded repeats, ultimately affecting gene transcription and repeat instability.

## 1. Introduction 

Over twenty unstable and expanded microsatellite repeats have been identified as the cause of human neurological disorders. These repeats, mostly consisting of trinucleotides or tetranucleotides, are considered dynamic mutations; they possess the unusual characteristic that repeat tract length is variable. Most microsatellite repeats show a normal range of relatively short and stable repeats and disease-causing longer tracts that are often unstable. Although differences exist between diseases, some molecular mechanisms overlap. A longer repeat is typically associated with more clinical problems, on top of earlier onset of symptoms. Since mutation rate increases with repeat length, successive generations are faced with larger risks of developing more severe disease, a phenomenon called anticipation [1, 2].

Myotonic dystrophy type I (DM1) is caused by an expanded CTG-repeat in the 3′ UTR of the *DMPK* gene that is quite unstable when transmitted to the next generation [3–5]. Myotonic dystrophy type I is a multisystem disorder with patients showing not only muscle problems, but also cataract, cardiac anomalies, testicular atrophy, gastrointestinal, and endocrine abnormalities, as well as problems originating in the central nervous system. Ongoing somatic expansion in DM1 patients is thought to contribute to disease progression [6].

In addition to DM1, many other trinucleotide repeat (TNR) diseases are highly debilitating. Efforts are therefore aimed at understanding not only pathogenesis, but also mechanisms of TNR instability. Thus far, replication, (bidirectional) transcription, and DNA repair processes have been described to play a role in TNR instability mechanisms [7–9].

One of the major pathogenic models proposed to underlie DM1 is a toxic effect of the presence of expanded CUG-containing transcripts. Mutant *DMPK* mRNAs are retained in the nucleus, accumulate in foci [10], and form complexes with regulatory proteins, thereby preventing these proteins from exerting their normal function [11]. Aberrant miRNA metabolism has also been described in patients with expanded CTG-repeats [12, 13]. Recent evidence that bidirectional transcription [14] and nonconventional RNA translation [15] are taking place at several TNR loci is complicating the traditional picture of RNA toxicity. These findings point at a scenario where not just one single expanded RNA transcript is responsible for disease development [11].

Moreover, chromatin dynamics are increasingly recognised to influence both TNR instability and gene expression at TNR loci and thereby probably disease outcome. TNRs can affect nucleosome positioning [16, 17], and CTG-repeats specifically have been identified as preferential location for nucleosome formation [18]. CTG- and CAG-repeats have been described to form a functional component of insulator elements, thereby influencing gene expression levels. At the DM1 locus, the CTG-repeat forms an insulator together with the two CTCF-binding sites (CTCFbs) that flank the repeat [19]. Long CTG-tracts were shown to induce condensation of DNA at the DM1 locus, which could hinder access of gene regulators to this region [20]. Transcription of the *SIX5* gene that neighbours *DMPK* was decreased in patient cells expressing expanded CTG-repeats [21]. These findings together led to the proposal that the expanded CTG-repeat induces a transcriptionally repressive region [21]. Indeed, long CTG-repetitions were shown to induce heterochromatin formation, which can then spread into neighbouring regions [22].

Aberrant chromatin remodelling has also been observed at other TNR disease loci. For instance, an expanded CGG-repeat is associated with CpG hypermethylation, heterochromatinisation, and silencing of the *FMR1* gene, which is the cause of Fragile X syndrome [23]. DNA methylation and histone modifications representative of silent chromatin have been observed around the expanded GAA-repeat in intron 1 of the *Frataxin* gene that causes Friedreich's ataxia [9].

Although the chromatin context is now considered important for DM1 [14] and other TNR loci [9, 23–25], few comprehensive studies addressing multiple factors involved in or associated with chromatin remodelling simultaneously have been performed. Recently, studies in mouse models for Huntington's disease suggested that the chromatin context of the transgene integration site determines CAG-repeat instability and transcription levels [26]. Thus, these processes seem tightly linked, underscoring the need to understand chromatin dynamics at TNR loci.

We therefore set out to study the consequences of CTG-repeat expansion for CpG methylation, CTCF-binding, chromatin conformation, and gene expression at the DM1 locus, making use of the transgenic mouse model previously generated in our laboratory [27]. These mice carry a large human genomic transgene that encompasses the *DMPK* gene as well as the neighbouring genes *DMWD* and *SIX5*. The transgene includes either a normal CTG-repeat of 20 trinucleotides or disease-associated expanded repeats, with the latter showing CTG-repeat instability patterns similar to DM1 patients (strongly biased towards expansions, length- and age-dependent somatic instability, albeit showing smaller repeat length changes per instability event in mice) [28–30]. The transgene also encompasses important regulatory sequences such as the two CTCF-binding sites (CTCFbs) that flank the CTG-repeat in humans and the enhancer of the downstream *SIX5* gene.

The phenotype of homozygous mice carrying up to 1600 CTGs has recently been characterised [31]. Since these mice display multiple characteristics seen in DM1 [27, 28, 32, 33], they are considered a valuable model to investigate mechanisms implicated in CTG-repeat instability and DM1 pathogenesis [34, 35]. Benefitting from this DM1 mouse model, we aimed to study the epigenetic consequences of the expanded CTG-repeat at the DM1 locus. We present evidence that expanded CTG-repeats induce CpG methylation and local heterochromatinisation and concurrent decreased transcription around the repeat, without affecting significantly CTCF binding at the DM1 locus. We also found binding of PCNA around the CTG-repeat and propose that it might lie at the basis of CTG-repeat expansion-induced repressive changes in chromatin dynamics.

## 2. Materials and Methods

### 2.1. Transgenic Mice

Mice used in this study harbour a transgene consisting of 45 kb of human genomic DNA cloned from a DM1 patient and have been described previously (crossbred to >90% C57/BL6 background) [27, 28, 31]. Mice were genotyped by PCR amplification of tail DNA using oligonucleotide primers DMHR8 (5′-TGACGTGGATGGGCAAACTG-3′), DMHR9 (5′-AGCTTTGCACTTTGCGAACC-3′), and Dmm9 (5′-GCTTGTAACTGATGGCTGGG-3′), which amplify the endogenous murine *Dmpk *(DMHR8 and Dmm9) and the human transgene *DMPK* (DmHR8 and DMHR9) [36]. CTG-repeat length was determined by PCR amplification of DNA extracted from tail at weaning, with oligonucleotide primer 101 (5′-CTTCCCAGGCCTGCAGTTTGCCCATC-3′) and primer 102 (5′-GAACGGGGCTCGAAGGGTCTTGTAGC-3′), as described before [37], followed by electrophoresis of PCR products on a large 0.8% (w/v) agarose gel [36]. For the current paper, heterozygous mice with the following CTG-repeat lengths were used: DM20: mice of the DM20-949 line that carry the normal unexpanded human allele, DM300: mice of the DM300-328 line that currently have an average of 610 CTGs (range: 545–700), and DMSXL: mice of the DM300-328 line that have undergone large expansions and now carry alleles with over 1000 CTGs [28]. In this particular study, mice with ~1300–1600 CTG-repeats (mean 1435 CTGs) were used. Adult mice of 3–5 months of age were used for all experiments described in this study (mean age did not differ among the different genotype groups, as tested with Kruskal-Wallis. DM20: mean age: 4.4 months, DM300: mean age: 4.3 months, and DMSXL: 4.1 months, *P* = 0.634). We chose heart as a representative tissue for disease, which shows the highest *DMPK* expression levels in our mice [31]. Hearts were dissected, snap-frozen in liquid nitrogen, and stored at −80°C until use.

Animals were housed and cared for according to guidelines by the *French Council on Animal Care “Guide for the Care and Uses of Laboratory Animals” EEC86/609 *Council Directive—Decree 2001-131.

### 2.2. Bisulfite Sequencing

Methylation status of the sequences flanking the CTG-repeat was studied by bisulfite conversion of DNA isolated from adult hearts (extracted with Qiagen DNeasy Blood and Tissue Kit, according to manufacturer's instructions).

500 ng of DNA was bisulfite-converted with Qiagen's Epitect Bisulfite kit. Bisulfite converts an unmethylated cytosine (C) into a thymine (T), while leaving methylated Cs unchanged. Subsequent PCR amplification and sequencing of the PCR product and comparison with the target (genomic DNA) sequence then allow distinction between Cs that were or were not methylated at the time of bisulfite conversion. Primers were chosen so as not to contain any CpGs, such that DNA templates can be amplified irrespective of their methylation status. Methylation interference studies identified guanine nucleotides whose methylation prevents binding by CTCF, predominantly on the noncoding strand [19]. As most DNase I-hypersensitive sites induced by CTCF binding are on this strand, we conclude that the status of this strand is most relevant for our studies. Therefore, we directed our CpG methylation studies at the non-CTG strand. Approximate amplicon locations can be seen in Figure 2(a). Seminested PCR was performed with the following primers for CTCFbs1: F (forward): 5′-TAGTAGTAGTAGTATTTT-3′, R1 (reverse): 5′-TAGTAGTAGTAGTATTTT-3′, and R2 (for seminested PCR in combination with primer F): 5′-CTTTCCCTACTCCTATT-3′. CTCFbs2 was amplified with F1: 5′-GTTTTGGGTAGATGGAGGGTT-3′, R: 5′-AATCACAAACCATTTCTTTCT-3′, and F2: 5′-GGTTTTAGGTGGGGATAGATA-3′. Three parallel seminested PCR reactions were performed with 4 *μ*L of PCR product (total reaction volume 25 *μ*L) of the first amplification as input, with an annealing time 2°C higher than the one used in the first PCR and 3 more cycles (30 and 33 cycles for subsequent PCR rounds), to obtain sufficient PCR product. PCR products were loaded onto an 1.5% (w/v) agarose gel. Products were subsequently cut out and snap-frozen in liquid N_2_ in columns of the Millipore DNA gel extraction kit. The snap-frozen agarose band was spun down and DNA in the flow-through was precipitated using classical NaCl and ethanol precipitation. These purified PCR products were used to subclone into pMosBlue vector, using the pMosBlue blunt-ended PCR cloning kit, according to manufacturer's instructions (GE Healthcare). Colony PCR was performed with primers T7 and U19 and correctly sized clones were sent for sequencing at the Sequencing Platform of Cochin Hospital in Paris, France. Sequences and CpG methylation of individual CpGs were subsequently analysed using BiQ Analyser software [38]. Of each mouse, at least 10 clones were sequenced. An average percentage of methylation per CpG was calculated per mouse, of which an overall weighted average percentage methylation was calculated per CpG, per genotype.

### 2.3. Chromatin Immunoprecipitation (ChIP) and Quantitative PCR (qPCR)

#### 2.3.1. Chromatin Preparation

Approximately 30 mg of frozen tissue was cut into small pieces. 500 *μ*L of cold PBS with 1% formaldehyde was added. Protein-DNA interactions were cross-linked for 10 minutes at room temperature (RT), while rotating. Fixation was quenched by adding glycine to a final concentration of 0.125 M and rotating for 5 minutes at RT. Samples were spun at ~470 g (2500 rpm in table top centrifuge) for 10 minutes at 4°C, with slow deceleration. The pellet was washed with cold PBS, for 10 minutes at 4°C, setting the centrifuge to slowly decelerate. Pellet was resuspended, vigorously vortexed in 150 *μ*L SDS lysis buffer (1% SDS, 10 mM EDTA, 50 mM Tris, pH 8, with PhosStop phosphatase inhibitors (Roche) and complete protease inhibitors (Roche)), and left on ice for 15 minutes, with repeated vortexing. The tissue samples were then sonicated (Branson Sonifier cell disruptor B15) in lysis buffer until macroscopically homogenised, while being kept cold. Samples were spun down at 11000 rpm, for 10 minutes at 4°C (normal deceleration from here onwards). 100 *μ*L of supernatant was transferred to a separate tube and kept on ice. 100 *μ*L of SDS lysis buffer was added to the pellet, in which the pellet was resuspended and vortexed. These samples were snap-frozen in liquid nitrogen twice to aid further lysis. Samples were centrifuged at 11000 rpm, for 10 minutes at 4°C. The supernatant was taken and added to the first supernatant fraction. The supernatant fraction was again sonicated to shear the chromatin, obtaining fragments of 200–1000 bp. The sheared chromatin was diluted 5 times with ChIP dilution buffer (0.01% SDS, 1.1% Triton X-100, 1.2 mM EDTA, 16.7 mM Tris-HCl pH 8.1, and 167 mM NaCl), supplemented with phosphatase and protease inhibitors.

#### 2.3.2. Chromatin Immunoprecipitation (ChIP)

40 *μ*L of Dynal protein A Dynabeads (Life technologies) per immunoprecipitation (IP) reaction was incubated with antibody at RT for one hour, while rotating. The following antibodies were used for ChIP: rabbit anti-CTCF (Abcam ab70303, 2 *μ*g per IP), rabbit anti-H3K9/14Ac (Millipore 06-599, 5 *μ*g), mouse anti-H3K27Me (Abcam ab6002, 2 *μ*g), rabbit anti-H3K9Me3 (Millipore 07-442, 3 *μ*g), and mouse anti-PCNA (Santa Cruz sc56, 5 *μ*g). 200 *μ*L of 5x diluted chromatin was taken per IP and subjected to a further 2x dilution with ChIP dilution buffer supplemented with phosphatase and protease inhibitors and added to the antibody-covered Dynabeads (the abChIP reaction). For each sample, a control IP was taken along, using rabbit or mouse IgG (Santa Cruz) as appropriate. An aliquot of the same cross-linked and sheared chromatin was kept aside and purified in parallel, to be used as input chromatin control for PCR. Antibody-covered beads were incubated with the chromatin overnight at 4°C, while rotating. The following day, the beads were washed, using a Dynal Dynamag-Spin Magnet (Life technologies), for 4 minutes at 4°C, while rotating, twice with ChIP dilution buffer, 1x with low salt immune complex washing buffer (0.1% SDS, 1% Triton X-100, 2 mM EDTA, 20 mM Tris-HCl pH 8.1, and 150 mM NaCl), 2x with high salt immune complex washing buffer (0.1% SDS, 1% Triton X-100, 2 mM EDTA, 20 mM Tris-HCl pH 8.1, and 500 mM NaCl), 1 time with LiCl immune complex buffer (0.25 M LiCl, 1% Igepal-CA630, 1% deoxycholic acid (sodium salt), 1 mM EDTA, 10 mM Tris pH 8.1, Millipore 20-156), and twice in TE buffer (10 mM Tris-HCl, 1 mM EDTA pH 8.0). Beads were then taken up in 150 *μ*L complete elution buffer (20 mM Tris-HCl pH 7.6, 5 mM EDTA, 50 mM NaCl, 1% SDS, and 130 *μ*g/mL proteinase K (Life technologies)), and incubated at 67°C for at least 4 hours, while rotating, to elute the protein-DNA complexes off the beads, reverse cross-links, and digest proteins simultaneously. Eluates were then collected, and beads were rinsed with 75 *μ*L elution buffer (20 mM Tris-HCl pH 7.6, 5 mM EDTA, and 50 mM NaCl). Eluates were combined and purified with Qiaquick DNA purification columns (Qiagen 28106), according to protocol, with the exception of the addition of 1 volume of isopropanol, in addition to 5 volumes of buffer PB in the first step to optimise isolation of small fragments. DNA was eluted in two steps using twice 25 *μ*L elution buffer provided with the kit.

#### 2.3.3. Quantitative PCR (qPCR)

**Figure 1:**
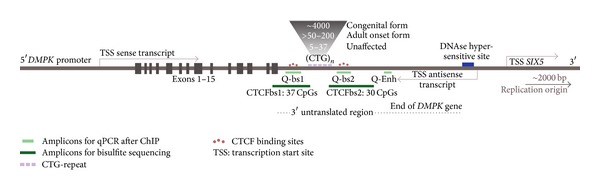
DM1 locus: schematic drawing of the DM1 locus, indicating relevant sites and regions, including the expanded CTG-repeat with flanking CTCF-binding sites (CTCFbs), the DNAse hypersensitive site enhancer of *SIX5*, and transcription start sites (TSS) of the genes located at the DM1 locus. Approximate locations of amplicons used for bisulfite sequencing and qPCR after ChIP are indicated. The 45 kb fragment of genomic DNA that was used to generate the DM300 transgenic mouse line used in this study contains all of the features indicated in this scheme.

Relative abundance at target loci in the ChIPed DNA was analysed using quantitative PCR, using Power SYBR green master mix (Applied Biosystems) in an AB7300 real-time PCR system (Applied Biosystems). For all antibodies used, amplicons were measured at CTCF binding site 1 (Q-bs1), CTCF binding site 2 (Q-bs2), and the enhancer region (Q-Enh), as indicated in Figure 1. Primer sequences were as follows: Q-bs1-Forward (F): 5′-CTGCCAGTTCACAACCGCTC-3′, Q-bs1-Reverse (R): 5′-CGAGCCCCGTTCGCCG-3′, Q-bs2-F: 5′-CGTCCGTGTTCCATCCTC-3′, Q-bs2-R: 5′-CGTCCGTGTTCCATCCTC-3′, Q-Enh-F: 5′-GGAGGCGTGTGGAGGCGG-3′, and Q-Enh-R: 5′-TCCCCCAACCCTGATTCG-3′. The following amplicons were used as positive or negative PCR controls for ChIP reactions: Myc-F: 5′-CTTGTTCTATTGCCTTTCCGTTTC-3′ and Myc-R: 5′-AACCCATCCCTACTTTCTGACAGTC-3′ (positive control for CTCF-ChIP), Gapdh-F: 5′-ATAAGCAGGGCGGGAGGC-3′ and Gapdh-R: 5′-CGTCTCTGGAACAGGGAGGAG-3′ (positive control for active histone mark, negative control for repressive histone modifications and CTCF), Amylase-F: 5′-CTCCTTGTACGGGTTGGT-3′ and Amylase-R: 5′-AATGATGTGCACAGCTGAA-3′ (negative control for active histone modification, positive control for PCNA), and HOXd9-F: 5′-TGCTCCGGGGCTTTGGATAA-3′ and HOXd9-R: 5′-CTCTCTGGGTCCTGCGATCT-3′ (positive control for repressive histone modifications). A standard curve of serial dilutions of genomic DNA was always taken along. A dissociation curve was run in every experiment to assess quality of the reaction and ensure absence of primer-dimer or other nonspecific PCR products. Making use of the formula derived of the standard curve, quantities were calculated from the obtained quantification cycle (*C*_*q*_) values for each sample. Each sample was performed in triplicate, and quantities were averaged. Quantities obtained in IP reactions (antibody-ChIP “abChIP” or IgG-mock IP “IgG-IP”) were normalised by division by the quantity obtained in input chromatin (non-IPed) samples (enrichment). Enrichments were normalised against the abChIP enrichment value of the positive control amplicon, for each respective CTG-repeat length category, to correct for possible differences in chromatin density. All pairs of normalised IgG- and abChIP-enrichment values for a given genotype and amplicon were subjected to a Mann-Whitney *U* test to see if enrichment in abChIP was statistically significantly larger than in IgG-IP, according to expectations (expected for positive control amplicons but not for negative control amplicons). The input-corrected enrichment values of all specific abChIP samples were further subjected to statistical analysis using IBM SPSS Statistics Standard Edition version 20 Software, to analyse possible effects of increasing CTG-repeat length. Graphs and statistical tests were obtained with GraphPad Prism version 5.0c. All effects are reported at a 0.05 level of significance.

### 2.4. Expression Studies

#### 2.4.1. RNA Isolation

Snap-frozen adult hearts taken from DM20, DM300, and DMSXL mice were homogenized in Trizol (Life Technologies) using a tissue lyser (Retsch MM400, 2x 2,5 minutes, using 2 stainless steel beads (Qiagen)). After chloroform extraction, the aqueous phase was mixed with 70% ethanol and transferred to a Spin Cartridge of the PureLink RNA Mini Kit (Ambion by Life Technologies). Total RNA was extracted according to manufacturer's instructions. A PureLink DNAse step was inserted in the protocol, as recommended after binding of the RNA to the column. RNA concentrations were determined by absorbance at 260 nm using a NanoDrop 1000 spectrophotometer (ThermoFisher Scientific) and quality and absence of genomic DNA were verified on agarose gel.

Different RNA extraction methods have been compared, of which the one described here was found to give the highest yield. Efficacy of the various methods to recover expanded RNA was assessed by comparing the total RNA recovery and *DMPK* expression levels between hemizygous and homozygous mice [31].

#### 2.4.2. Reverse Transcriptase (RT) PCR

cDNA was synthesized from 0.4–0.6 *μ*g RNA, using Superscript II Reverse Transcriptase (Life Technologies) when using random hexamer primers (for *SIX5* mRNA quantification) or using Superscript III Reverse Transcriptase (Life Technologies) when using strand-specific primers (for *DMPK* sense and antisense mRNA quantification), according to manufacturer's instructions (also see [31]). cDNA was treated with RNAse A for 20 minutes at 37°C. Please refer to Table 1 for primer sequences used for strand-specific RT.

#### 2.4.3. Quantitative RT-PCR (qRT-PCR)

Relative abundance of transcripts was analysed using Power SYBR green master mix (Applied Biosystems) in an AB7300 real-time PCR system (Applied Biosystems). Annealing temperatures and sample dilutions were optimized for each amplicon. *DMPK*, *SIX5*, and antisense transcript levels were calculated relative to *18s* and endogenous murine *Dmpk* mRNA levels. Oligonucleotide primer sequences are described in Table 1. For qRT-PCR we used standard curves of serial dilutions of a plasmid carrying the amplicon. Reverse transcriptase efficiency for each gene and each primer set was verified using increasing amounts of RNA as input. A dissociation curve was run in every experiment to assess quality of the reaction and ensure absence of primer-dimer or other nonspecific PRCR products. Reverse transcriptase was performed in duplo, followed by separate qPCR analyses on each cDNA sample. All qPCR reactions were performed in triplicate and experiments (from RT reaction to qPCR analysis) were done twice. RNA from hearts of 8 DM20, 5 DM300, and 6 DMSXL mice was used for these expression studies. Averages of triplicate quantities obtained for each mouse were normalised against a control sample that was taken along in every qPCR experiment. The average expression level of the two parallel qRT-PCR experiments was subjected to statistical analyses. Jonckheere Terpstra test for trend (IBM SPSS Statistics Standard Edition version 20 Software) was performed to investigate whether expression levels change with increasing CTG-repeat length. Differences between repeat length categories were further investigated by means of non-parametric Mann-Whitney pairwise comparisons. Graphs and statistical tests were obtained with GraphPad Prism version 5.0c. All effects are reported at a 0.05 level of significance.

## 3. Results

### 3.1. Elevated CpG Methylation with Increasing CTG-Repeat Length

**Figure 2:**
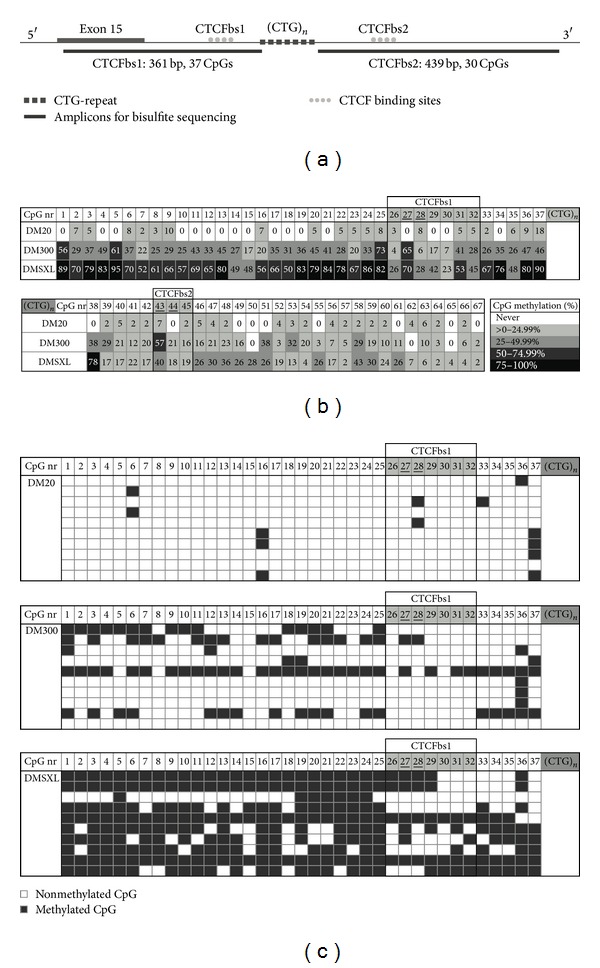
CpG methylation increases with CTG-repeat length. (a) Schematic drawing of the position of the amplicons obtained with seminested PCR, used for bisulfite sequencing, relative to both CTCF-binding sites, the CTG-repeat and exon 15, in the 3′ region of the *DMPK* transgene. (b) CpG methylation in hearts of adult mice increases with expanding CTG-repeat length. For each repeat length category, 4 mice were used, and per mouse at least 10 clones were sequenced after bisulfite conversion. CpGs are numbered from 5′ to 3′. Shaded CpG numbers lie within the CTCF-binding site recognition sequence. Underlined CpG numbers are CpG dinucleotides that contain G residues essential for recognition by CTCF [19]. Numbers indicate the weighted average percentage of methylation at a particular CpG, seen across all clones in all 4 mice. Colour coding further indicates the approximate degree of methylation at a given CpG. (c) Example of methylation profiles obtained in different clones, representing different cardiac cells of 1 mouse per repeat length category. Individual clones show substantially distinct methylation patterns.

We studied CpG methylation around the CTG-repeat, since DNA methylation can affect binding of transcription factors or attract chromatin-remodelling enzymes. Previous methylation analyses [39] had shown substantial CpG methylation in various tissues of DM300 mice, in both upstream and downstream regions flanking the CTG-repeat, while DM20 tissues were almost completely devoid of CpG methylation. CpG methylation analysis in DM1 patient tissues showed a clearly polarised pattern, with only methylated Cs at and around CTCFbs1 and not at CTCFbs2. Substantial variability between individual patients can be observed [39]. We extended these observations by performing a detailed analysis of the CpG methylation pattern by bisulfite sequencing in individual mice carrying normal alleles with 20 CTGs (DM20), or mice expressing expanded alleles (DM300: 545–700 repeats or DMSXL: 1300–1600 CTGs). We studied the region flanking the CTG-repeat as shown in Figure 1. We chose to study heart, which shows the highest *DMPK* expression levels in our mice [31]. Heart abnormalities including arrhythmias and conduction defects are a central feature to the disease. Bisulfite sequencing was directed at the non-CTG strand, because a methylation interference assay indicated that CTCF showed stronger contacts on this strand [19]. Approximate amplicon locations used in this study are indicated in Figure 2(a).

Figure 2(b) illustrates the results of 10 clones (representing 10 different cells) of four mice for each repeat length category. It shows that CpG methylation is very low in DM20 mice, more prominent in DM300 mice, and quite abundant in DMSXL mice. Further expansion of the CTG-repeat from around 600 to ~1450 CTGs is associated with more pronounced CpG methylation. This trend is seen around both CTCFbs1 and CTCFbs2, although less pronounced at CTCFbs2. Overall, the region around CTCFbs1 is more methylated than the region around CTCFbs2. Note that CTCFbs1 itself is relatively spared from CpG methylation, as compared to its flanking sequences.  Figure 2(c), illustrating the methylation pattern of each clone, shows that the CpG methylation pattern is variable in individual cells within a tissue. Other mice that we analysed showed a similar pattern of distinct methylation patterns in individual cells (data not shown).

### 3.2. CTCF Still Binds CTCF-Binding Sites When CTG-Repeat Is Expanded

Based on *in vitro* observations, it had been postulated that binding of CTCF is lost when CpGs in the CTCF recognition sequence are methylated or mutated [19]. Since we did not observe an all-or-nothing CpG methylation pattern, we investigated CTCF binding to the two CTCFbs flanking the CTG-repeat. We performed ChIP on chromatin preparations of adult heart, comparing the three repeat length categories, followed by qPCR to analyse quantities of immunoprecipitated DNA. Positions of amplicons at the DM1 locus are drawn in Figure 1. For ChIP analyses of histone modifications and CTCF binding we performed control experiments with both a positive and a negative control amplicon. Overall, these control experiments led to satisfactory results. Details of statistical analyses can be found in Supplementary Tables 1 and 2 (see Supplementary Material available online at http://dx.doi.org/10.1155/2013/567435).

**Figure 3:**
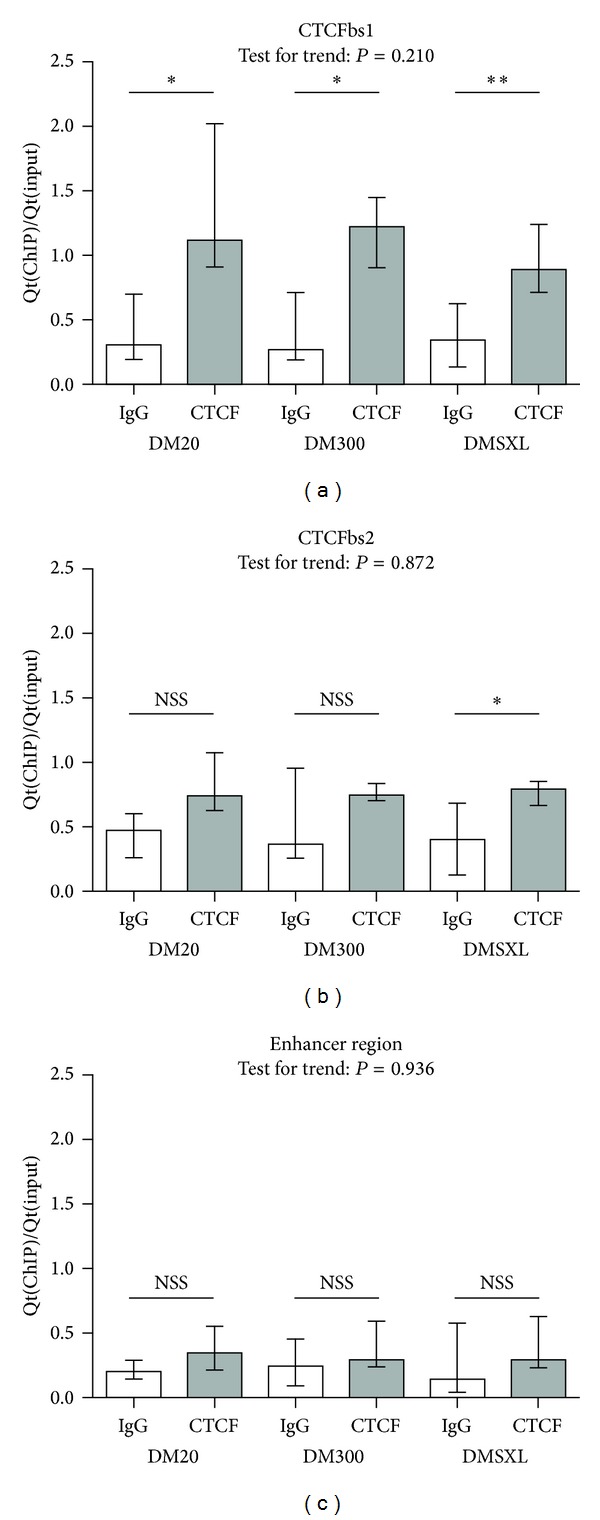
CTCF binds to CTCFbs1, also in the presence of expanded CTG-repeats. These graphs show enrichment ((Qt(IP)/Qt(input), normalised against the abChIP enrichment value of the positive control amplicon of each respective repeat length category) for CTCF in abChIP versus IgG-IP at three regions at the DM1 locus. CTCFbs1 shows statistically significant enrichment for CTCF in abChIP versus IgG-IP in all repeat length categories. No such CTCF-binding is seen at CTCFbs2 or at the enhancer region. Height of the bars indicates median enrichment; error bars indicate the interquartile range (25th to 75th percentile of observations). Mann-Whitney tests were performed to test for a statistically significant difference between the abChIP and IgG reactions. Results are summarised here with * being *P* < 0.05, ** being *P* < 0.01, and *** being *P* < 0.001. Details of the statistical analysis can be found in Supplementary Table 1. Enrichment values obtained for abChIP reactions were subjected to the Jonckheere Terpstra test for trend, which tests whether a trend exists across the categories with increasing CTG-repeat length. *P* values are indicated in the graphs and details of this statistical analysis can be found in Supplementary Table 2.

We observed enrichment in CTCF-immunoprecipitated samples of all CTG-repeat length categories at CTCFbs1, but no significant difference between mice carrying normal or expanded repeat (Figure 3). Although the enrichment appeared slightly lower in DMSXL as compared to DM20 and DM300, this trend did not reach statistical significance.

At CTCFbs2, binding of CTCF seemed lower than binding at CTCFbs1 and enrichment was significant only in DMSXL mice, probably due to experimental variability. No significant trend across the categories was observed for CTCF enrichment at CTCFbs2.

At the enhancer region, no statistically significant enrichment was seen, showing, as expected, no CTCF binding in this region (Figure 3).

### 3.3. CTG-Repeat Expansion Is Associated with Local Chromatin Remodelling around the CTCFbs

Methylated CpGs may attract chromatin-remodelling enzymes; thus we analysed chromatin remodelling in the presence of an expanded CTG-repeat in our mice. We performed ChIP with antibodies directed against histone modifications that represent actively transcribed (H3K9/14Ac) or repressed (H3K27Me3 and H3K9Me3) chromatin. Enrichment for these histone modifications around CTCFbs1, CTCFbs2, and the enhancer region was studied by qPCR on chromatin immunoprecipitated DNA. Approximate amplicon locations at the DM1 locus are indicated in Figure 1.

#### 3.3.1. H3K9/14Ac: Histone Modification Representative of Transcriptionally Active Chromatin

**Figure 4:**
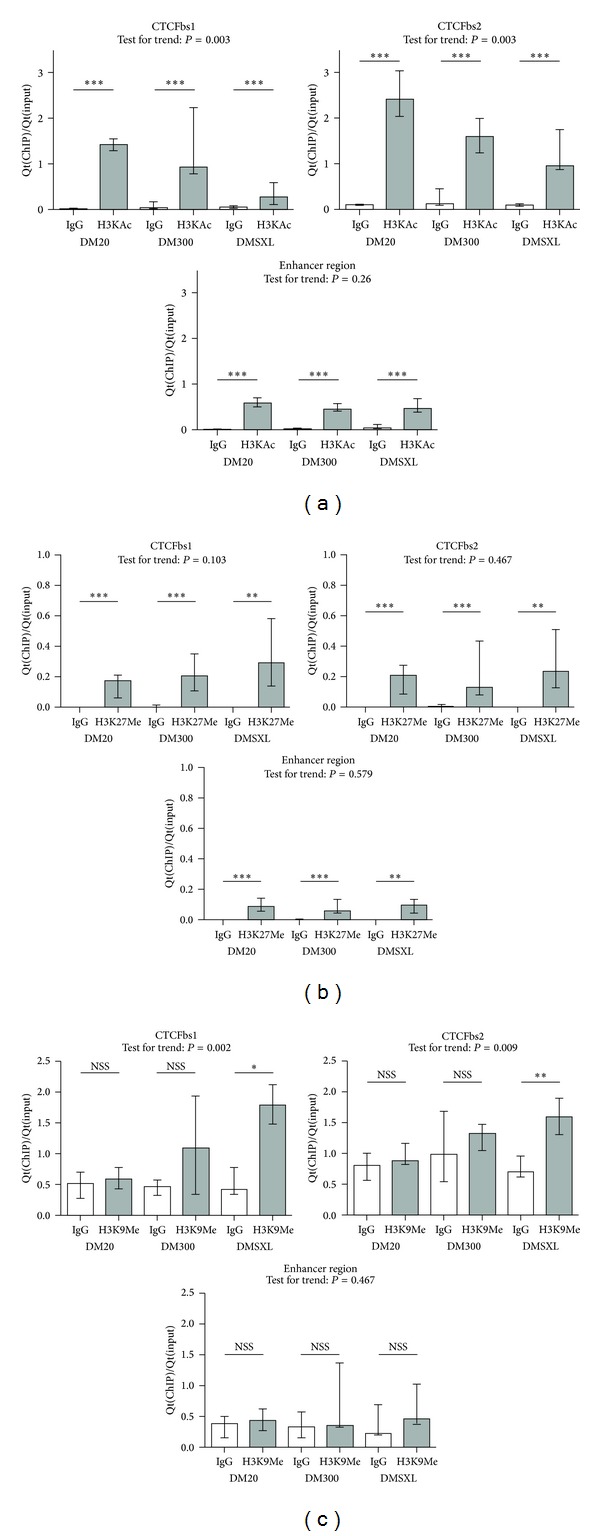
Expanded CTG-repeats are associated with local chromatin remodelling towards a less active chromatin conformation around the CTG-repeat. Less occupancy of active histone marks H3K9/14Ac (H3KAc in graph) (a) and more repressive histone marks (H3K9Me3) (c) around CTCFbs1 and bs2 is observed upon CTG-repeat expansion, while enrichment of repressive mark H3K27Me3 (b) is unaffected. These graphs show enrichment (Qt(IP)/Qt(input), normalised against the abCHIP enrichment value of the positive control amplicon of each respective repeat length category) for different histone modifications in abChIP versus IgG-IP at three regions at the DM1 locus. Height of the bars indicates the median enrichment, and error bars indicate the interquartile range (25th to 75th percentile of observations). Mann-Whitney tests were performed to test for a statistically significant difference between the abChIP and IgG reactions. Results are summarised here with * being *P* < 0.05, ** being *P* < 0.01, and *** being *P* < 0.001. Details of the statistical analysis can be found in Supplementary Table 1. The enrichment values obtained for the specific abChIP reactions were subjected to the Jonckheere Terpstra test for trend, which tests whether a trend exists across the categories with increasing CTG-repeat length. *P* values are indicated in the graphs and details of this statistical analysis can be found in Supplementary Table 2.

We observed statistically significant enrichment with an antibody directed against acetylated H3K9/14 (H3K9/14Ac) around CTCFbs1, CTCFbs2, and to a lower extend at the enhancer region (Figure 4(a)). At both CTCFbs1 and bs2, Jonckheere Terpstra test for trend revealed a statistically significant decrease of H3K9/14Ac enrichment across CTG-repeat length categories (CTCFbs1: *z*-score: −2.931, *P* = 0.003, CTCFbs2: *z*-score: −2.996, *P* = 0.003, where a negative *z*-score indicates a descending trend; thus a lower median enrichment was seen with increasing CTG-repeat length). Hence, chromatin of mice with longer repeats was less enriched for the active histone modification than chromatin of control mice. Enrichment at the enhancer region was low and did not show a trend across the different categories of mice.

#### 3.3.2. H3K27Me3 and H3K9Me3: Histone Modifications Indicative of Transcriptionally Repressed Chromatin

As a first investigation of possible heterochromatinisation, we performed ChIP with an antibody directed against the repressive histone mark trimethylated H3K27 (H3K27Me3). We saw low but statistically significant enrichment at all amplicons and in all CTG-repeat length categories, as shown in Figure 4(b). Although enrichment appeared slightly higher in the DMSXL mice for the CTCFbs1 and CTCFbs2 amplicons, no statistically significant trend with increasing repeat length was seen.

We next investigated another histone methylation mark representative of transcriptionally repressed chromatin: trimethylated H3K9 (H3K9Me3, Figure 4(c)). DM20 and DM300 did not show statistically significant enrichment in the specific antibody ChIP reaction (abChIP) versus IgG-IP at CTCFbs1 and bs2, whereas DMSXL did. Thus, H3K9Me3 only binds at and around the CTCFbs in DMSXL samples. This was confirmed by a statistically significant trend across categories (CTCFbs1: *z*-score: 3.084, *P* = 0.002, CTCFbs2: *z*-score: 2.599, *P* = 0.009). No statistically significant enrichment nor a statistically significant trend for H3K9Me3 enrichment was seen at the enhancer region (Figure 4(c)).

### 3.4. Lower *DMPK* Sense and *SIX5* Transcript Levels in Mice with Expanded CTG-Repeats, While Antisense Transcript Levels Appear Unaffected by CTG-Repeat Length

**Figure 5:**
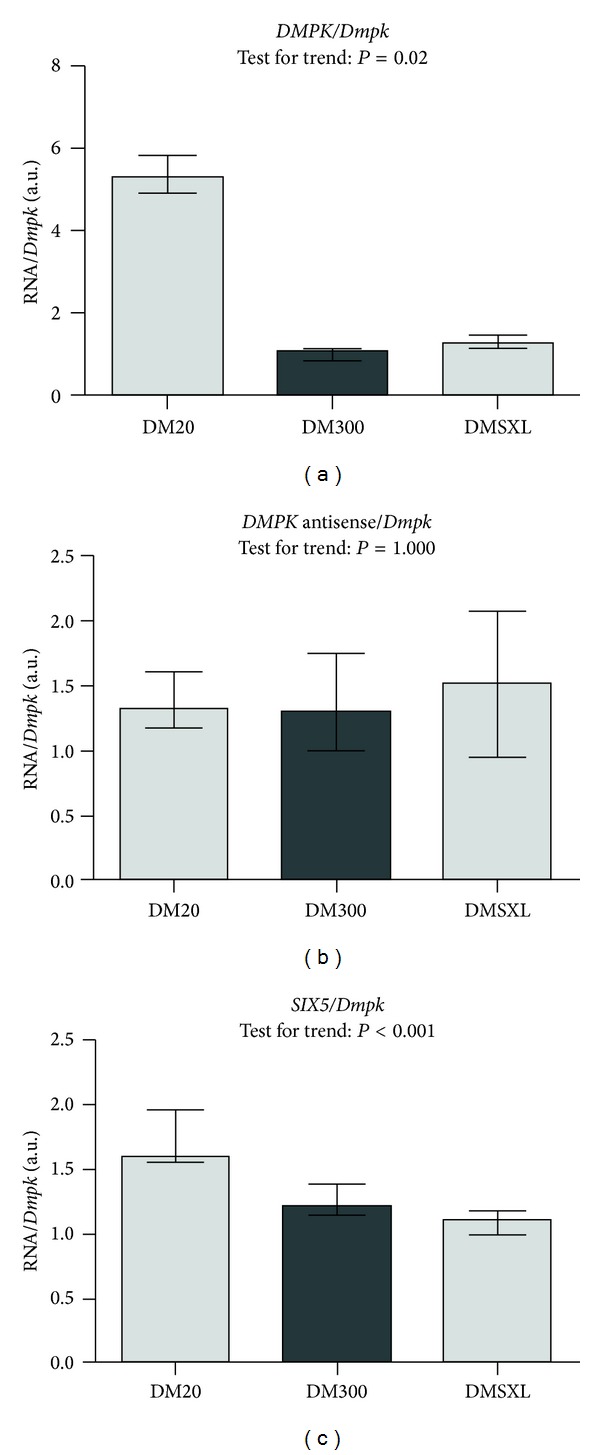
Decreased sense transcript levels at the DM1 locus in the presence of expanded CTG-repeats. Upon CTG-repeat expansion, decrease of *DMPK* sense and *SIX5* transcript levels is observed, while *DMPK* antisense levels are unaffected by CTG-repeat length. These graphs show relative abundance (in arbitrary units) of transcripts of interest, corrected by two different reference genes (*18s* (a) and endogenous murine *Dmpk* (b)). Height of bars indicates the median enrichment, and error bars indicate the interquartile range (25th to 75th percentile of observations). Relative abundance values were subjected to the Jonckheere Terpstra test for trend, which tests whether a trend exists across the categories with increasing CTG-repeat length. *P* values are indicated in the graphs and details of this statistical analysis can be found in Supplementary Table 3.

We investigated possible changes in expression levels at the DM1 locus, since chromatin remodelling is generally accompanied with changes in gene expression. Sense *DMPK *transcript levels showed a sharp decrease between DM20 and mice with expanded repeats (Figure 5). An overall statistically significant trend was observed across the repeat length categories for both reference genes (see Supplementary Table 3 for results of Jonckheere Terpstra test for trend: normalised against *18s*: *z*-score: −3.160, *P* = 0.002, *Dmpk*: *z*-score: −2.332, *P* = 0.02). Mann-Whitney pairwise comparisons did not show a statistically significant decrease in *DMPK* mRNA expression between DM300 and DMSXL (medians and interquartile ranges are shown in Supplementary Table 3, data of Mann-Whitney analysis not shown). The *DMPK* antisense transcript did not show a similar trend of changing expression across repeat length categories when antisense mRNA levels were normalised against *Dmpk* (Figure 5) (*Dmpk*: Jonckheere Terpstra test for trend *z*-score: 0, *P* = 1, Supplementary Table 3). However, a statistically significant trend was observed when antisense transcript levels were normalised against *18s* (*z*-score: −2,107, *P* = 0.035). Post hoc pairwise Mann-Whitney comparisons however did not show statistically significant differences between any of the repeat length categories, when correcting the *P* values for multiple comparisons (data not shown).

*SIX5* expression levels are affected by increasing repeat length, as they show a significant decrease across the repeat length categories. The decrease is lower than that seen for *DMPK *sense transcripts, but consistent with both reference genes (Jonckheere Terpstra test for trend: versus *18s*: *z*-score: −2.332, *P* = 0.02, versus *Dmpk*: *z*-score: −3.912, *P* < 0.001, Supplementary Table 3). No difference was observed between DM300 and DMSXL mice.

### 3.5. PCNA Binding Near the Expanded CTG-Repeat

PCNA (proliferating cell nuclear antigen) is involved in many cellular processes [40]. In addition to its role in replication, PCNA recruits a variety of epigenetic regulators [41]. Loops of slipped-strand structures formed by expanded CTG-repeats could serve as loading sites for PCNA and binding of PCNA to the expanded CTG-repeat could be the beginning of the cascade of chromatin remodelling event [8, 42].

**Figure 6:**
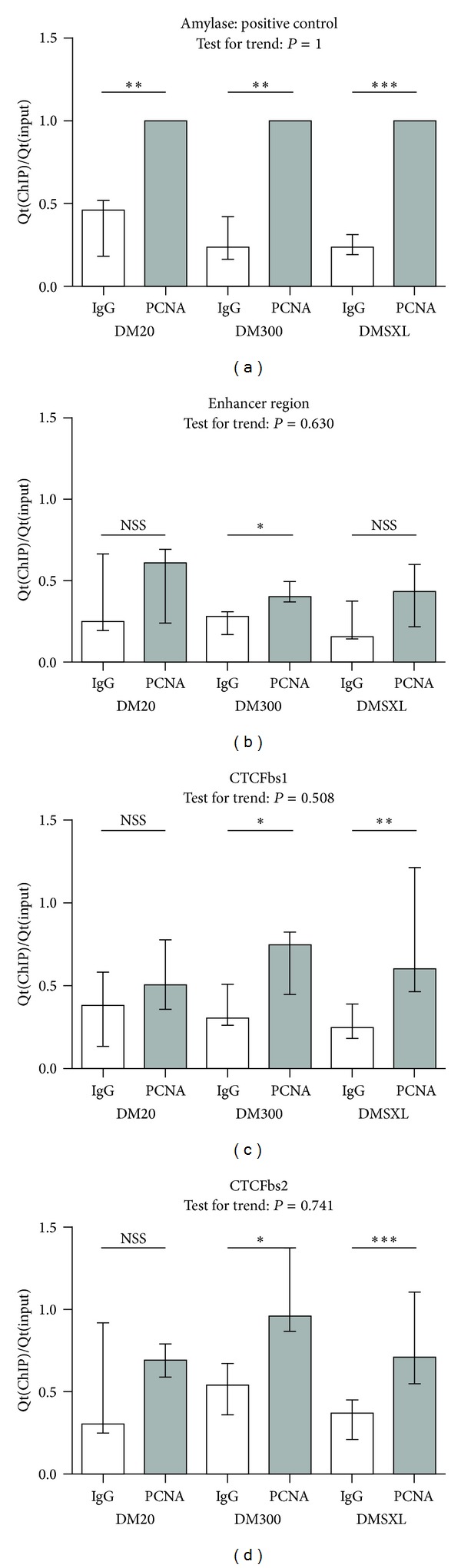
PCNA seems to bind to expanded CTG-repeats. CTCFbs1 and bs2, but not the enhancer region, show statistically significant enrichment of PCNA in abChIP versus IgG-IP in the expanded CTG-repeat length categories, but not at DM20, suggesting that PCNA binds the expanded CTG-repeat. These graphs show enrichment (Qt(IP)/Qt(input), normalised against the abCHIP enrichment value of the positive control amplicon of each respective repeat length category) for PCNA in abChIP versus IgG-IP at a positive control amplicon and three regions at the DM1 locus. Height of the bars indicates the median enrichment, and error bars indicate the interquartile range (25th to 75th percentile of observations). Mann-Whitney tests were performed to test for a statistically significant difference between the abChIP and IgG reactions. Results are summarised here with * being *P* < 0.05, ** being *P* < 0.01, and *** being *P* < 0.001. Details of the statistical analysis can be found in Supplementary Table 1. The enrichment values obtained for the specific abChIP reactions were subjected to the Jonckheere Terpstra test for trend, which tests whether a trend exists across the categories with increasing CTG-repeat length. *P* values are indicated in the graphs and details of this statistical analysis can be found in Supplementary Table 2.

We therefore investigated whether PCNA binds near the CTG-repeats in our mice, by ChIP, followed by qPCR analysis. Enrichments measured with the enhancer amplicon were modest compared to the positive *Amylase* control and were significant only for DM300 mice (*P* = 0.01, IgG versus PCNA antibody, Figure 6). At both CTCFbs1 and 2, we did not see statistically significant enrichment at DM20, but enrichment was detected for DM300 and DMSXL, suggesting binding of PCNA to expanded CTG-repeats. Jonckheere Terpstra test for trend did not reveal a statistically significant trend, indicating that similar PCNA-binding was detected despite a longer repeat. At the enhancer region, on the contrary, we did not observe statistically significant enrichment, except for modest enrichment in DM300. Thus, these preliminary data seem to suggest that PCNA specifically binds close to the amplicons at CTCFbs1 and 2, but not the enhancer region.

## 4. Discussion

We here show that expanded CTG-repeats induce a locally repressed chromatin state and accompanying reduced sense gene transcription at the DM1 locus in adult transgenic mouse hearts.

Mice with expanded repeats showed substantial methylation at and around the CTCFbs, as opposed to DM20, which showed very little CpG methylation. This CpG methylation is not seen at all CpGs nor in all individual cells, but overall DNA methylation levels are higher with increasing repeat length. DM20 and DM300/SXL are independent transgenic lines and we cannot exclude an influence of the transgene integration site sequences. However, the transgene is large (45 kb) and contains the major regulatory sequences between *DMPK* and *SIX5* [14, 19]. All different lines obtained with different repeat lengths showed the same tissue-specific pattern of *DMPK* expression, which is also similar to the mouse *Dmpk* gene and to the *DMPK* gene in human tissues [31, 32]. In addition, expression levels correlate to copy number of the integrated transgene indicating that the surrounding mouse sequences have no or minimal impact on the transgene.

We observed that the percentage of cells carrying a methyl group at a given CpG was substantial in DM300 and even higher in DMSXL, both at CTCFbs1 and bs2. CpG methylation was more abundant at CTCFbs1 than at CTCFbs2, confirming a polarised localisation of methylation at the DM1 locus as described before [39]. This is in line with evidence pointing at a more important regulatory role for CTCFbs1 [19, 43]. Our observation that the CTCFbs1 recognition sequence itself was relatively spared from CpG methylation as opposed to surrounding sequences is also worth noting in this respect.

CpG methylation around the DM1 CTG-repeat had previously been assessed by measuring the height of chromatogram peaks obtained for cytosine after sequencing of bisulfite-converted DNA [39]. That study showed differences in methylation patterns between DM300 mice and humans [43]. Most importantly, adult human DM1 samples never showed methylation at CTCFbs2, whereas DM300 mice did, indicating that the mouse model does not fully mimic the human situation underlining limitations of animal models. However, one human foetus did show CpG methylation at and around CTCFbs2, indicating individual variation among human patients [39]. We demonstrate here that mice with around 1450 CTGs clearly show more methylation than mice with around 600 CTG with a strong bias 5′ of the CTG-repeat. Mice with longer expanded repeat tracts may therefore better mimic the human situation.

The variable DNA methylation pattern found in our mice around the CTG-repeat resembles that observed around the SCA7-CAG-repeat in a transgenic mouse model for Spinocerebellar Ataxia 7 (SCA7) [24]. A strong correlation between the severity of disease symptoms and level of DNA methylation has been described at the CGG-repeat and promoter region of the *FMR1* gene [44, 45]. In addition, it was recently proposed that also variable methylation patterns in the promoter of the *ATXN2* gene explain considerable variation in anticipation, in the absence of intergenerational CAG-repeat instability. Different degrees of methylation of the *ATXN2* promoter could be related to age of onset in patients with SCA2 SCA3, suggesting that gene dosage through this epigenetic mechanism is important for disease outcome [46]. Thus, these observations underline that CpG methylation is no all-or-nothing phenomenon at TNR loci and underscore the importance of careful examination of methylation status of individual CpGs. Relevant mechanistic information might be missed when a more general approach is followed.

*In vitro* studies have shown disrupted binding of CTCF to the CTCFbs upon mutation or methylation of the recognition sequence [19]. Since our data show that CpG methylation is no all-or-nothing phenomenon at the studied locus, it was unclear what to expect concerning CTCF binding. For the first time in mammalian tissues we show that CTCF still binds to CTCFbs1 despite the presence of an expanded CTG-repeat of up to ~1600 units (Figure 3). We did not detect significant binding of CTCF to CTCFbs2, which is consistent with *in vitro* binding assays that showed stronger binding of CTCF to site 1 [19]. It was surprising to observe clear CTCF binding at CTCFbs1 *in vivo* despite abundant CpG methylation of the region. However, it is interesting to note that the CTCF recognition sequence is relatively spared from methylation when compared to the adjacent region. It is possible that the repeat in our mice is not large enough to induce full methylation of the binding site. Alternatively, at the *H19* locus, binding of CTCF has been demonstrated to prevent CpG methylation [47, 48]. Further research may shed light on the order of events.

Methylated CpGs are known to attract chromatin-remodelling enzymes [41, 49, 50]. *In vitro,* the nucleosome assembly of DNA containing repeating CTG triplets showed that the efficiency of nucleosome formation increased with expanded triplet blocks [16–18], suggesting that such blocks may profoundly alter local chromatin structure and repress transcription through the creation of stable nucleosomes. We therefore explored possible chromatin rearrangement in mice with expanded CTG-repeats, as opposed to DM20. We found chromatin remodelling indicative of a transcriptionally repressed state, close to the expanded CTG-repeat, in DM300 and DMSXL mice (Figure 4). The enhancer region generally showed a different enrichment pattern for histone modifications than around the CTCFbs, suggesting that a local region of heterochromatin is formed close to the expanded CTG-repeat, within a euchromatin region. This has previously been demonstrated in patient cells [14]: heterochromatin spreading was seen upon expansion of the CTG-repeat, as active HK4Me3 was replaced by the repressive H3K9Me3 mark. When the expanded CTG-repeat induced heterochromatinisation, adjacent genes were silenced by propagation of heterochromatin along the chromosome [22]. We did see increased enrichment of H3K9Me3 around very long CTG-repeats, but heterochromatinisation did not propagate to the enhancer region. Prominent decreasing trends of H3K9/14Ac enrichment at both CTCFbs were observed across increasing CTG-repeat length categories. A similar graded loss of acetylated H3 and H4 with increasing CGG-repeat length has been observed in FXS patient cells [23]. Considering that qPCR reactions for CTCFbs1 and bs2 were performed on the same ChIPed DNA and that qPCR efficiencies were very similar, it might be concluded that CTCFbs2 is more enriched for H3K9/14Ac. As yet, we cannot know whether this has a functional implication or whether it is linked to the seemingly more important regulatory role of CTCFbs1.

We chose to study one active histone modification only, as genome-wide histone modification maps show that the distribution of most histone marks recognised to be active is highly similar [51]. Fewer histone modifications associated with repressive chromatin have been described and little is known about their global linkage [51]. Our data show that H3K9Me3 and H3K27Me3 did not respond in the same way to the expanded CTG-repeat. In a study that investigated the epigenetic status of the euchromatic region of the human Y chromosome, H3K9Me3 and H3k9Ac enrichment correlated with the expression status, whereas H3K27Me3 enrichment did not. This suggests a mechanism where H3K9Me3 and H3K9Ac dominate over H3K27Me modifications to determine expression status of the chromatin [52]. A similar situation seems to be the case in our adult mouse hearts.

Consistent with chromatin changes representative of transcriptional repression, we saw lower sense *DMPK* and *SIX5* expression in mice that carry an expanded CTG-repeat (Figure 5). We did not detect a further decrease of *DMPK* and *SIX5* transcription levels when comparing DM300 and DMSXL at 5 months of age, despite the 30% decrease previously observed at 2 months of age in the same transgenic mice [31]. This could be due to the decreased transgene expression we observed with age (data not shown). Therefore factors other than chromatin may also contribute to the change in DM1 expression.

The *DMPK *antisense transcript emanates from the *SIX5 *adjacent regulatory region. In DMSXL mice it is expressed in many tissues, with expression being the highest in heart, as is the case for the *DMPK* sense transcript, although it does not follow nor mirror the same expression profile [31]. Sense messenger levels are higher than antisense. Since the antisense transcript and *SIX5* have overlapping promoter regions, it could be postulated that they are subject to similar regulatory factors. However, in the current study,* SIX5 *mRNA levels decreased, whereas antisense transcript levels remained unaffected in the presence of an expanded CTG-repeat. Interestingly, this finding demonstrates that regulation of *DMPK* antisense is independent although some regulatory sequences might be shared with *DMPK* and *SIX5*. Future research will likely shed more light on the role of bidirectional transcription in the *DMPK* gene and at other TNR loci.

Previous studies have demonstrated that PCNA can be loaded onto dsDNA-ssDNA junctions in DNA-loops or loops of slipped-strand structures formed by expanded CTG-repeats [8, 42]. We therefore recognised in PCNA a candidate molecule that might bind to the expanded CTG-repeat and then cause a cascade of chromatin-modifying events. We here show evidence that PCNA indeed binds to or close to the expanded CTG-repeat (Figure 6). PCNA appears to bind to a similar extent to CTCFbs1 and bs2 amplicons, which is according to expectations, since both amplicons lie very close to the CTG-repeat. It is possible that more PCNA was bound to the longer CTG-repeat in DMSXL mice, but that we cannot detect this due to the size of our sheared fragments.

By recruiting epigenetic regulators, PCNA might be the instigator of multiple downstream chromatin modifications [41]. PCNA is known to interact with DNA methyltransferases DNMT1, -3A and -3B, as well as with histone methyltransferases. These interactions cause H3K9 and H3K27 to become trimethylated, yielding a repressed chromatin environment. Via another route, DNMT1 interacts with histone deacetylases (HDACs), which also contributes to a repressed chromatin context [41]. The observed enrichment pattern of histone modifications and hypermethylation around the expanded CTG-repeat in adult hearts of mice fit with this model. Importantly, we detected enrichment for PCNA in mice with expanded CTG-repeats, to a similar extent at both CTCFbs, but not at the enhancer region, suggesting that PCNA binds to or very near the expanded CTG-repeat specifically.

DNA methylation and histone modifications appear to reciprocally influence each other [41]. Thus, multiple parallel pathways seem responsible for the establishment of a repressed chromatin status.

The involvement of PCNA needs to be confirmed and the precise order of events remains to be elucidated.

We here presented evidence that expanded CTG-repeats induce CpG methylation and local heterochromatinisation close to the repeat. This is accompanied by decreased levels of sense *DMPK* and *SIX5* transcription. CTCF binding at the DM1 locus was not affected by the expansion of the CTG-repeat. We found that PCNA binds in the vicinity of expanded CTG repeats and might be recruited to the expanded CTG-repeat. We propose that it could subsequently attract chromatin-remodelling enzymes that yield the repressive changes in chromatin dynamics. A better understanding of the precise cascade of processes induced by expanded TNRs, and, importantly, the starting point of these changes, will provide us with therapeutic targets to alleviate disease progression and limit further TNR expansion.

## Supplementary Material

This supplementary material consists of three tables with details of the statistical analyses of the chromatin immunoprecipitation (ChIP) experiments and expression analyses.
For the ChIP experiment, supplementary table 1 shows median enrichment values of each IgG-abChIP pair (corrected for the positive control value) and the U-statistic and P-value of the Mann-Whitney test that was performed to test if abChIP differed significantly from IgG. Table 2 gives all values for median enrichment and interquartile range (25^th^ and 75^th^ percentile), which were subjected to the Jonckheere-Terpstra test for trend. Z-score and P-values of this test for trend are given in supplementary table 2 as well. Supplementary table 3 gives the median values and interquartile range of relative abundance of transcripts of interest, and the details on the Jonckheere-Terpstra test for trend that was performed on the expression data.

## Figures and Tables

**Table 1:** List of primers used for expression analysis.

*DMPK* sense	
primer for RT:	CGACTGGAGCACGAGGACACTGACTTGCTCAGCAGTGTCAGCAGGTCCCCGCC
qPCR primers:	Forward: CGACTGGAGCACGAGGACACTGA
	Reverse: GGAGAGGGACGTGTTG
*DMPK* antisense	
primer for RT:	CGACTGGAGCACGAGGACACTGAGACCATTTCTTTCTTTCGGCCAGGCTGAGGC
qPCR primers:	Forward: GGAGCACGAGGACACTGA
	Reverse: TGCGAACCAACGATAG
*SIX5 *	
primer for RT:	Random hexamers
qPCR primers:	Forward: TGGTGGTGCTGGGGGTTGTATC
	Reverse: GGGGCAGGGTGTTCCGCTTAC
*Dmpk* (specific priming)	
primer for RT:	CGACTGGAGCACGAGGACACTGACTCAGCAGCGTTAGCA
qPCR primers:	Forward: GGAAGAAAGGGATGTATTA
	Reverse: CTCAGCAGCGTTAGCA
*Dmpk* (random priming)	
primer for RT:	Random hexamers
qPCR primers:	Forward: GGAAGAAAGGGATGTATTA
	Reverse: CTCAGCAGCGTTAGCA
*18S* (specific priming)	
primer for RT:	CGGGTTGGTTTTGATCTG
qPCR primers:	Forward: CAGTGAAACTGCGAATGG
	Reverse: CGGGTTGGTTTTGATCTG
*18S* (random priming)	
primer for RT:	Random hexamers
qPCR primers:	Forward: CAGTGAAACTGCGAATGG
	Reverse: CGGGTTGGTTTTGATCTG
*Gapdh* (specific priming)	
primer for RT:	TGTAGACCATGTAGTTGAGGTCA
qPCR primers:	Forward: AGGTCGGTGAACGGATTTG
	Reverse: TGTAGACCATGTAGTTGAGGTCA
*Gapdh* (random priming)	
primer for RT:	Random hexamers
qPCR primers:	Forward: AGGTCGGTGAACGGATTTG
	Reverse: TGTAGACCATGTAGTTGAGGTCA
